# Experimental and numerical investigations of the water surface profile and wave extrema of supercritical flows in a narrow channel bend

**DOI:** 10.1038/s41598-024-61297-8

**Published:** 2024-05-28

**Authors:** Subhojit Kadia, I. A. Sofia Larsson, Mats Billstein, Leif Lia, Elena Pummer

**Affiliations:** 1 Department of Civil and Environmental Engineering, Norwegian University of Science and Technology, 7491 Trondheim, Norway; 2 Division of Fluid and Experimental Mechanics, Luleå University of Technology, 97181 Luleå, Sweden; 3 Vattenfall AB, R&D Hydraulic Laboratory, 81470 Älvkarleby, Sweden

**Keywords:** Curved channel, Experimental study, Numerical simulation, OpenFOAM, Sediment bypass tunnel, Supercritical flow, Wave maxima and minima, Civil engineering, Computational science, Fluid dynamics, Power stations

## Abstract

Supercritical flows in channel bends, e.g., in steep streams, chute spillways, and flood and sediment bypass tunnels (SBTs), experience cross-waves, which undulate the free surface. The designs of these hydraulic structures and flood protection retaining structures in streams necessitate computing the locations and water depths of the wave extrema. This study numerically and experimentally investigates the water surface profiles along the sidewalls, the wave extrema flow depths, and their angular locations in a narrow channel bend model of the Solis SBT in Switzerland. The 0.2 m wide and 16.75 m long channel has a bend of 6.59 m radius and 46.5° angle of deviation. The tested flow conditions produced Froude numbers ≈ 2 and aspect ratios ranging from 1.14 to 1.83. Two-phase flow simulations were performed in OpenFOAM using the RNG *k–ε* turbulence closure model and the volume-of-fluid method. The simulated angular locations of the first wave extrema and the corresponding flow depths deviate marginally, within ± 6.3% and ± 2.1%, respectively, from the experimental observations, which signifies good predictions using the numerical model. Larger deviations, especially for the angular locations of the wave extrema, are observed for the existing analytical and empirical approaches. Therefore, the presented numerical approach is a suitable tool in designing the height of the hydraulic structures with bends and conveying supercritical flows. In the future, the model’s application shall be extended to the design of the height and location of retaining walls, embankments, and levees in steep natural streams with bends.

## Introduction

### Background

Natural streams and numerous hydraulic structures such as spillways, dam outlets, weirs, and flood and sediment bypass tunnels (SBTs) convey supercritical flows with Froude numbers Fr > 1.0. Their hydraulic designs can involve complex flow characteristics induced by secondary flows and cross-waves. Two kinds of secondary currents are observed in open channel flows. First, bend-induced secondary currents, or “secondary currents of Prandtl’s first kind”, are observed in curved channels due to the centrifugal force and the resulting radial pressure gradient^1–3^. The other kinds, turbulence-driven secondary currents, also known as “secondary currents of Prandtl’s second kind”^1–3^, are observed in open channels and non-circular ducts due to turbulence anisotropy and non-homogeneity induced by solid and free surface boundaries even if the channel is straight and uniform^3^. In narrow open channels with channel aspect ratios *a*_*r*_ = *b/h* ≤ 5.0, where *b* = channel width and *h* = flow depth, the turbulence-driven secondary currents and related velocity-dips are observed throughout the channel width^3–6^. They are also observed across the whole channel width due to difference in roughness between the bed and sidewalls^4,7,8^ and due to alternate bed roughness patches^8–11^. The secondary currents redistribute the high- and low-momentum fluids across the channel and undulate the lateral distribution of the bed shear stress, which can influence sediment transport^3–5,12–14^. Although the magnitude of the maximum secondary velocity for turbulence-driven secondary currents in narrow channels generally lies between 1.5 and 3% of the streamwise component^3,4,15–18^, such a quantity can reach as high as 20–30% in the case of bend-induced secondary currents^3^, which have a stronger effect on the flow characteristics than do the turbulence-driven secondary currents.

**Figure 1:**
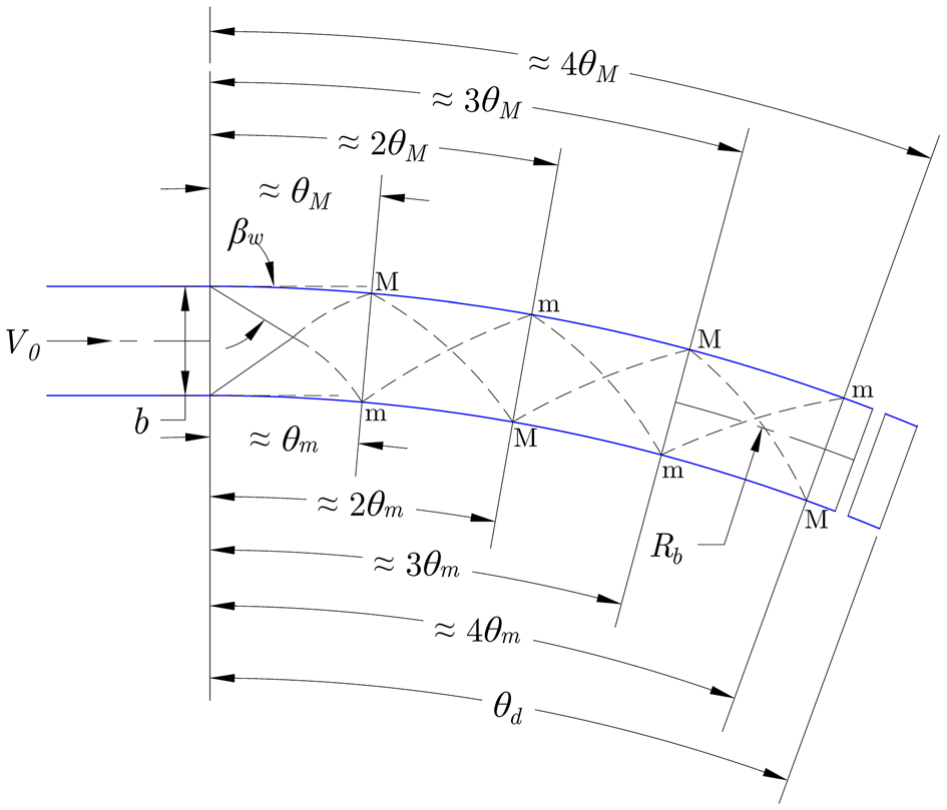
A schematic diagram showing the wave extrema characteristics in supercritical flow in a channel bend (after Reinauer and Hager^20^) [‘M’ indicates the wave maxima and ‘m’ indicates the wave minima].

In addition, in curved channels, the bend entrance acts as the source of flow disturbance that generates positive and negative cross-waves (also known as shock waves) from opposite banks, which propagate downstream in the case of supercritical flows^19^ with flow velocity greater than the wave velocity or the celerity. This results in alternate wave maxima and wave minima along the opposite banks as indicated by ‘M’ and ‘m’ in Fig. 1. The maximum flow depth is observed on the outer wall at the first wave maxima, which is useful in the design of channel (such as chute spillways) height and local rising of the outer wall or in the design of tunnel (such as SBTs) depth. Furthermore, the maximum flow depth and its angular location in steep river bends are useful in the hydraulic design of retaining walls, embankments, and levees to protect floodplains from being inundated. Therefore, accurate prediction of the wave maxima flow depth and position are crucial. In addition, for high Fr values, the flow cross-section becomes triangular and the inner wall dries as observed by Reinauer and Hager^20^ at Fr = 8. These phenomena are also influenced by the bend curvature.

The available design guidelines for SBTs suggest avoiding in-plan bends^21^, which can cause stronger secondary currents and significant lateral variations in bed shear stress, sediment transport, and invert abrasion. However, the project design and the geographical and geological conditions can make such bends unavoidable. Some existing SBT projects, e.g. the Asahi SBT in Japan^22^ and the Mud Mountain SBT in the USA^23^, have witnessed deeper invert abrasions toward the inner wall than toward the outer wall in SBT bends, and such effects have also continued in the downstream straight channels. Such observations reflect the requirement of localized specific design criteria. To understand the detailed flow characteristics, bed shear stress variation, bed load concentration, and water surface undulation characteristics of supercritical bend channel flows, an experimental investigation is being carried out by NTNU in co-operation with HydroCen, Vattenfall AB, Luleå University of Technology, and ETH Zürich. However, the present study is limited to the investigation of water surface undulations occurring along a channel bend through experiments and developing and validating an open-source numerical model.

### A review of previous experimental, analytical, and numerical studies

**Figure 2:**
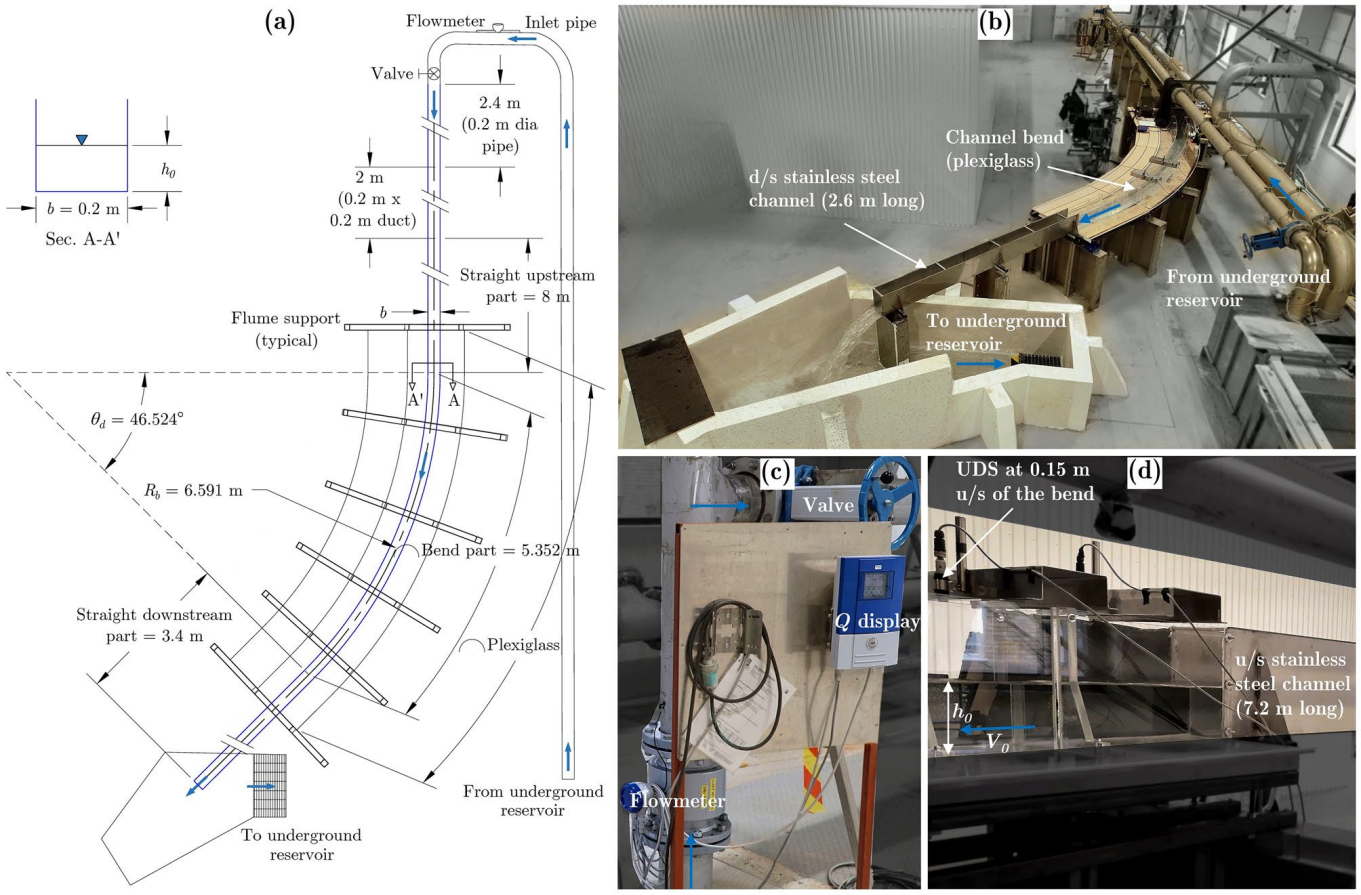
Experimental set-up: (**a**) schematic plan, (**b**) photographic view of the running model, (**c**) use of the electromagnetic flowmeter, and (**d**) use of an ultrasonic distance sensor (UDS) [u/s is upstream and d/s is downstream].

Table 1 summarizes the basic geometric and hydraulic parameters for the previous and present experimental studies dealing with supercritical flows in channel bends. Ippen^24^ and Ippen and Knapp^19^ were the primary investigations those explained the complex free surface undulations associated with supercritical flows in channel bends based on experiments conducted in rectangular channels with width *b* = 0.305 m, adjustable bed slope *S*_*b*_ between 1.45 and 9.95%, relative radii of curvature *r*_*c*_ = *b/R*_*b*_ = 0.1, 0.05, and 0.025 (where *R*_*b*_ = radius of the bend at the channel center as shown in Figs. 1 and 2a), and angles of deviation *θ*_*d*_ = 22.5° and 45°. It was observed that the cross-wave patterns continue in the straight channel downstream of the bend, and such patterns do not depend on *R*_*b*_ when the wave angle *β*_*w*_ remains constant for a constant *S*_*b*_. Furthermore, the specific energy diagram indicates that the disturbance caused by the wave is greater for flow conditions close to the critical flow (Fr = 1) where the specific energy change is heavily influenced by the flow depth, which influences the celerity. In continuation with those earlier studies, Knapp^25^ introduced the following simplified analytical approach for wave profiles:

1 $$Y = \frac{h}{{h_{0} }} = {\text{Fr}}^{2} \sin^{2} \left( {\beta_{w} \pm \frac{1}{2}\theta } \right);\beta_{w} = \tan^{ - 1} \left( {\left( {{\text{Fr}}^{2} - 1} \right)^{ - 1/2} } \right)$$

2 $$\theta_{0} = \tan^{ - 1} \left( {\frac{b}{{\left( {R_{b} + {\raise0.7ex\hbox{$b$} \!\mathord{\left/ {\vphantom {b 2}}\right.\kern-0pt} \!\lower0.7ex\hbox{$2$}}} \right)\tan \beta_{w} }}} \right) = \tan^{ - 1} \left( {\frac{{r_{c} \left( {{\text{Fr}}^{2} - 1} \right)^{1/2} }}{{\left( {1 + {\raise0.7ex\hbox{${r_{c} }$} \!\mathord{\left/ {\vphantom {{r_{c} } 2}}\right.\kern-0pt} \!\lower0.7ex\hbox{$2$}}} \right)}}} \right)$$

where *h* = flow depth on sidewalls, *h*_*0*_ = approach flow depth, ${\text{Fr}} = {{V_{0} } \mathord{\left/ {\vphantom {{V_{0} } {\sqrt {gh_{0} } }}} \right. \kern-0pt} {\sqrt {gh_{0} } }}$ = Froude number of the approach flow, *V*_*0*_ = approach flow velocity, *g* = gravitational acceleration, *θ*_*0*_ = angular location of the first wave extrema, *θ* = angular location on the walls, and (±) indicates the wave extrema where (+) signifies the wave maxima and (−) signifies the wave minima. This method is best suited for Fr > 1.5 but is not suitable for steep waves where wave breaking occurs. However, no upper limit for Fr was mentioned, and the limiting values for *r*_*c*_ are missing ^20^. Later, Reinauer and Hager ^20^ simplified the equations for large Fr and small curvature values as follows:

3 $$Y = {\text{Fr}}^{2} \sin^{2} \left( {{\text{Fr}}^{ - 1} \pm \frac{1}{2}\theta } \right);\beta_{w} \approx \tan \beta_{w} \approx \sin \beta_{w} = \frac{C}{{V_{0} }} = {\text{Fr}}^{ - 1}$$

4 $$Y_{e} = \frac{{h_{e} }}{{h_{0} }}\;\;\; = \left( {{1} \pm \frac{1}{2}r_{c} {\text{Fr}}^{2} } \right)^{2} = \left( {{1} \pm \frac{1}{2}B_{n}^{2} } \right)^{2} {\text{for }}B_{n} < \sqrt 2$$

where *C* = celerity = $\sqrt {gh_{0} }$, *h*_*e*_ = flow depth on the sidewalls at the corresponding wave extrema, and *B*_*n*_ = bend number = ${\text{Fr}}\sqrt {r_{c} }$. Reinauer and Hager^20^ proposed an empirical approach (Eqs. 5–10) to obtain the wave extrema flow depths (Eqs. 5 and 6) and their angular locations (Eqs. 7 and 8) and the water surface profiles (WSPs) along the sidewalls (Eqs. 9 and 10) based on previous and own experimental data. Their experiments were conducted for 0.25 to 0.5 m wide channels with *r*_*c*_ ranging from 0.07 to 0.31, *θ*_*d*_ = 30° and 51°, and Fr ranging from 2 to 12.

5 $$\frac{{h_{M} }}{{h_{0} }} = \left( {0.4B_{n}^{2} { + 1}} \right)^{2} {\text{ for }}B_{n} \le 1.5;\quad \frac{{h_{M} }}{{h_{0} }} = \left( {0.6B_{n} { + 1}} \right)^{2} {\text{ for }}B_{n} > 1.5$$

6 $$\frac{{h_{m} }}{{h_{0} }} = \left( {1 - 0.5B_{n}^{2} } \right)^{2}$$

7 $$\tan \theta_{M} = r_{c} {\text{Fr for }}r_{c} {\text{Fr}} \le 0.35;\;\tan \theta_{M} = 0.6\sqrt {r_{c} {\text{Fr}}} {\text{ for }}r_{c} {\text{Fr}} > 0.35$$

8 $$\tan \theta_{m} = \sqrt {2} \,r_{c} {\text{Fr }}$$

9 $$\psi_{M} = \frac{{h - h_{0} }}{{h_{M} - h_{0} }} = \left( {\sin \left( {\frac{\pi }{2}\frac{\theta }{{\theta_{M} }}} \right)} \right)^{1.5} {\text{ for }}{\theta \mathord{\left/ {\vphantom {\theta {\theta_{M} }}} \right. \kern-0pt} {\theta_{M} }} \le 1.25$$

10 $$\psi_{m} = \frac{{h - h_{0} }}{{h_{m} - h_{0} }} = 1 - \left( {\sin \left( {\frac{\pi }{2}\frac{\theta }{{\theta_{m} }}} \right)} \right)^{1.5} {\text{for }}{\theta \mathord{\left/ {\vphantom {\theta {\theta_{m} }}} \right. \kern-0pt} {\theta_{m} }} \le 1.2$$

where *h*_*M*_ = the first wave maxima flow depth on the outer wall, *h*_*m*_ = the first wave minima flow depth on the inner wall, and *θ*_*M*_ and *θ*_*m*_ are the angular locations of the first wave maxima on the outer wall and the first wave minima on the inner wall, respectively, as shown in Fig. 1. Furthermore, one needs to be careful of the scale effects associated with experimental studies, which are hindered by water depths above 0.03 m^20^. Reinauer and Hager^20^ defined weak bends and strong bends (when the water surface separates from the inner wall) as *B*_*n*_ < 1.5 and *B*_*n*_ > 1.5, respectively. Recently, Amara et al.^26^ presented an analytical model based on a quasi-2D (two-dimensional) approach, which provides an analytical solution of the 2D shallow water equations and can approximately calculate the WSPs along the outer wall in supercritical bend flows. However, the produced triangularly shaped WSPs deviate considerably from the experimental results. In addition, some details of the other experimental works are provided in Table 1. The present study yields lower *a*_*r*_, *r*_*c*_, and *B*_*n*_ values than did previous studies.

**Table 1:** Summary of the basic geometric and hydraulic parameters for the previous and present experimental works on supercritical flows in channel bends.

Researchers	*b* (m)	*S*_*b*_ (%)	*R*_*b*_ (m)	*θ*_*d*_ (deg)	*h*_*0*_ (m)	*V*_*0*_ (m/s)	Fr	*a*_*r*_ = *b/h*_*0*_	*β*_*w*_ (deg)	*r*_*c*_	*B*_*n*_
Beltrami et al. ^27^	0.2–0.3	1.2–3.3	0.8–1.5	180	0.026–0.06	1.14–2.43	1.95–3.26	3.85–8.33	17.9–30.8	0.13–0.25	0.91–1.54
Ippen ^24^; Ippen and Knapp ^19^	0.305	1.45–9.95	3.05–12.2	22.5 and 45	0.015–0.095	1.42–4.32	2.33–6.89	3.21–20.84	8.3–25.4	0.025–0.1	0.37–2.17
Marchi ^28^	0.1–0.203	NA	0.05–0.4	90	0.021–0.061^a1^	0.83–2.1^a1^	1.14–3.7^a1^	3.28–9.39^a1^	15.7–61.5^a1^	0.5–2.0	0.8–5.21^a1^
Poggi ^29^	0.25	5–10	3.0–6.25	30 and 45	NA	NA	2.3–5.1	NA	10.9–19.1	0.04–0.083	0.57–1.47
Reinauer and Hager ^20^	0.25–0.5	NA	1.62–3.61	30 and 51	0.03–0.05	1.4–6.51^a^	2–12	5–16.67	4.8–30	0.07–0.31	0.66–4.55
Tian et al. ^30^	0.3	2	1.63	60	0.143–0.161	1.79–3.15	1.52–2.5	1.86–2.1	23.5–41.3	0.18	0.65–1.07
Present study	0.2	1.9	6.59	46.5	0.109–0.175	2.06–2.71	≈ 2	1.14–1.83	28.9–31.7	0.03	0.33–0.36

NA, not available.

^a^Calculated from *h*_*0*_ and Fr; ^a1^from the experimental sets A_2_, B, C, and E of Marchi ^28^.

The complex WSPs in supercritical channel flows have also been studied numerically, which can be an alternate quick and economical design solution to experimental studies. Ellis and Pender^31^ initiated a numerical study on the WSPs of supercritical flows in channel bends using 2D shallow water equations while neglecting the influences of the channel bed slope and friction. Later, Ellis^32,33^ found good agreement between the predicted and observed results after incorporating those influences into the initial model. Other researchers, such as Berger and Stockstill^34^, Valiani and Caleffi^35^, and Ghaeini-Hessaroeyeh et al.^36^, have also predicted WSPs using 2D shallow water equations. Ghaeini-Hessaroeyeh et al.’s^36^ predictions for small relative curvature *b/R*_*b*_ while assuming hydrostatic pressure and negligible diffusion were subsequently improved by Ghazanfari-Hashemi et al.^37^, who used commercial Ansys-Fluent to perform three-dimensional (3D) simulations of WSPs and wave characteristics for three cases from Poggi’s experiments^29^. Although Ghazanfari-Hashemi et al.^37^ reported that the selection of a turbulence model does not have any significant effect on the computed results, this statement was further analyzed in the present study. Previously, Ye et al.^38^ investigated the hydraulic characteristics of the S-shaped spillway in the Xiaonanhai reservoir via experiments and 3D numerical simulation and found that the numerical simulations were useful for such spillway designs. Brown and Crookston^39^ used the commercial computational fluid dynamics (CFD) software Flow-3D to simulate eight cases from Ippen^24^ and observed satisfactory agreement between the experimental and numerical WSPs along walls for milder channel slopes, but found significant deviations for higher Fr and steeper channels, especially along the inner wall. Huang and Wang^40^ simulated the 3D bend channel flow of an existing steep chute spillway using Flow-3D and obtained WSPs those are comparable to field data. Numerical model studies suggest using 3D simulation for proper estimation of complex cross-wave characteristics and water surface undulations.

### Objective of the study

The objective of this study is to investigate the water surface profiles along the sidewalls, the wave extrema flow depths, and their angular locations in a narrow channel bend model flowing under supercritical flow conditions via laboratory experiments and numerical simulation performed using the open-source CFD software OpenFOAM. Furthermore, we compare those results with the existing analytical and empirical approaches to check their applicability. To achieve these goals, three tests were performed both experimentally and numerically for discharges *Q* = 0.045 m^3^/s, 0.07 m^3^/s, and 0.095 m^3^/s; approach flow depths *h*_*0*_ = 0.109 m, 0.151 m, and 0.175 m; aspect ratios *a*_*r*_ = 1.83, 1.32, and 1.14; and Froude numbers Fr ≈ 2. These tested *a*_*r*_ and Fr values are comparable to that observed in existing SBTs^17^.

## Materials and methods

### Experimental work

A physical scale model with a scale factor of 1:22 representing the downstream bend part of the Solis SBT was built in the R&D Hydraulic Laboratory (hall 80, shown in Fig. 2) of Vattenfall AB at Älvkarleby, Sweden. The channel is 0.2 m wide, 0.3 m deep, and 16.75 m long (central length) and has an average *S*_*b*_ = 1.9% and comprises a bend with *θ*_*d*_ = 46.5°, *R*_*b*_ = 6.59 m, and *r*_*c*_ = 0.03. The curved channel or bend part (5.35 m curved length along the center, as shown in Fig. 2a) and 0.8 m straight upstream and downstream parts are made of 0.01 m thick transparent plexiglass to allow visual measurements. The remaining 7.2 m upstream and 2.6 m downstream straight parts were made of 0.002 m thick stainless-steel plates. Figure 2a shows a schematic plan view of the experimental set-up and Fig. 2b–d show experimental arrangements in the laboratory. The approach flow depth *h*_*0*_ was measured at a section 0.15 m upstream of the bend, as shown in Fig. 2d. It was obtained by averaging the flow depth measurements acquired across the channel at 0.025 m intervals. The flow depths on the walls were acquired using an angular scale (make Hultafors) with a least count of 0.001 m, and the flow depths away from the walls were acquired using Ultrasonic Distance Sensor (UDS) of make Micro Detectors (model UK1A/E2-0E). These UDS measurements were acquired at a frequency of 500 Hz for approximately 60 s using NI LabView (version 20.0.1) software. The data were subsequently postprocessed in MATLAB, version R2021a.

An underground reservoir was used to circulate the flow and the discharge was regulated by adjusting the pump load with a remote control and using the inlet valve shown in Fig. 2c. A pre-calibrated electromagnetic flowmeter of make Krohne (model Optiflux 4000) with ± 0.2% accuracy was placed at an inlet pipe 0.2 m in diameter (see Fig. 2c) to measure the discharge. To regulate the inlet flow, a transition from circular pipe flow to open channel flow was performed upstream of the steel channel section using a 2.0 m long square duct with a cross-section of 0.2 m × 0.2 m, as indicated in Fig. 2a. Once the flow was initiated or the discharge was changed, it took a few minutes before the flow stabilized, as observed from the discharge fluctuations found from the flowmeter; therefore, a waiting period of 10–15 min was required before taking any measurements. During the measurement, the free surface was fluctuating by a couple of mms. Therefore, angular scale measurements were taken with careful observation of such fluctuations over a period of 15–30 s to minimize measurement uncertainties. The measurements were taken up to the first two wave cycles at intervals of 0.1 m, with exceptions close to the wave extrema locations, where 0.05 m intervals were considered when 0.1 m intervals were not sufficient to obtain the crest or trough. Double-sided measuring tapes (as shown later in section “Comparison between the experimental, numerical, analytical, and empirical results”) with a 0.001 m least count were attached on the inner side of the plexiglass sidewalls to locate the bend entrance and the transformed (degree to meter) angular location of the bend on the outer and inner walls. The bend entrance refers to 0.8 m tape measurement on the outer wall and 10.8 m tape measurement on the inner wall. The average temperature of the water was found to be 16.6 °C.

The scale effects are negligible in the present study, as the flow depths and Reynolds numbers ${\text{Re}} = {{V_{0} D_{h} } \mathord{\left/ {\vphantom {{V_{0} D_{h} } \nu }} \right. \kern-0pt} \nu }$ (provided in Table 2), where *D*_*h*_ = hydraulic diameter and *ν* = kinematic viscosity of water, satisfy the recommended minimum values of *h* = 0.03 m^20^ and 0.04 m^41^ and of Reynolds numbers = 10^5^^42^ for supercritical flows. Furthermore, the approach flow conditions are comparable to those of recent experimental studies performed for supercritical flows by Auel et al.^5^, Jing et al.^43^, and Demiral et al.^12^.

**Table 2:** Hydraulic parameters and grid arrangements for the simulated cases.

Case number	Case name	*Q* (m^3^/s)	*h*_*0*_ (m)	Re (× 10^5^)	Fr	Turbulence model	Cell arrangements (longitudinal × lateral × vertical)			Total number of cells	Simulation execution time (minutes)
							Upstream	Bend	Downstream		
1	Q70 or Q70_8mm or Q70_RNG	0.07	0.151	5.12	≈ 2.0	RNG *k–ε*	315 × 26 × 39	337 × 26 × 39	63 × 26 × 39	725,010	94
2	Q70_6mm	0.07	0.151	5.12		RNG *k–ε*	400 × 32 × 48	428 × 32 × 48	80 × 32 × 48	1,394,688	279
3	Q70_10mm	0.07	0.151	5.12		RNG *k–ε*	250 × 21 × 31	268 × 21 × 31	50 × 21 × 31	369,768	29
4	Q70_ke	0.07	0.151	5.12		*k–ε*	315 × 26 × 39	337 × 26 × 39	63 × 26 × 39	725,010	90
5	Q70_koSST	0.07	0.151	5.12		*k–ω* SST	315 × 26 × 39	337 × 26 × 39	63 × 26 × 39	725,010	94
6	Q45	0.045	0.109	3.95		RNG *k–ε*	315 × 26 × 28	337 × 26 × 28	63 × 26 × 28	520,520	52
7	Q95	0.095	0.175	6.34		RNG *k–ε*	315 × 26 × 39	337 × 26 × 39	63 × 26 × 39	725,010	112

### Numerical simulation using OpenFOAM

**Figure 3:**
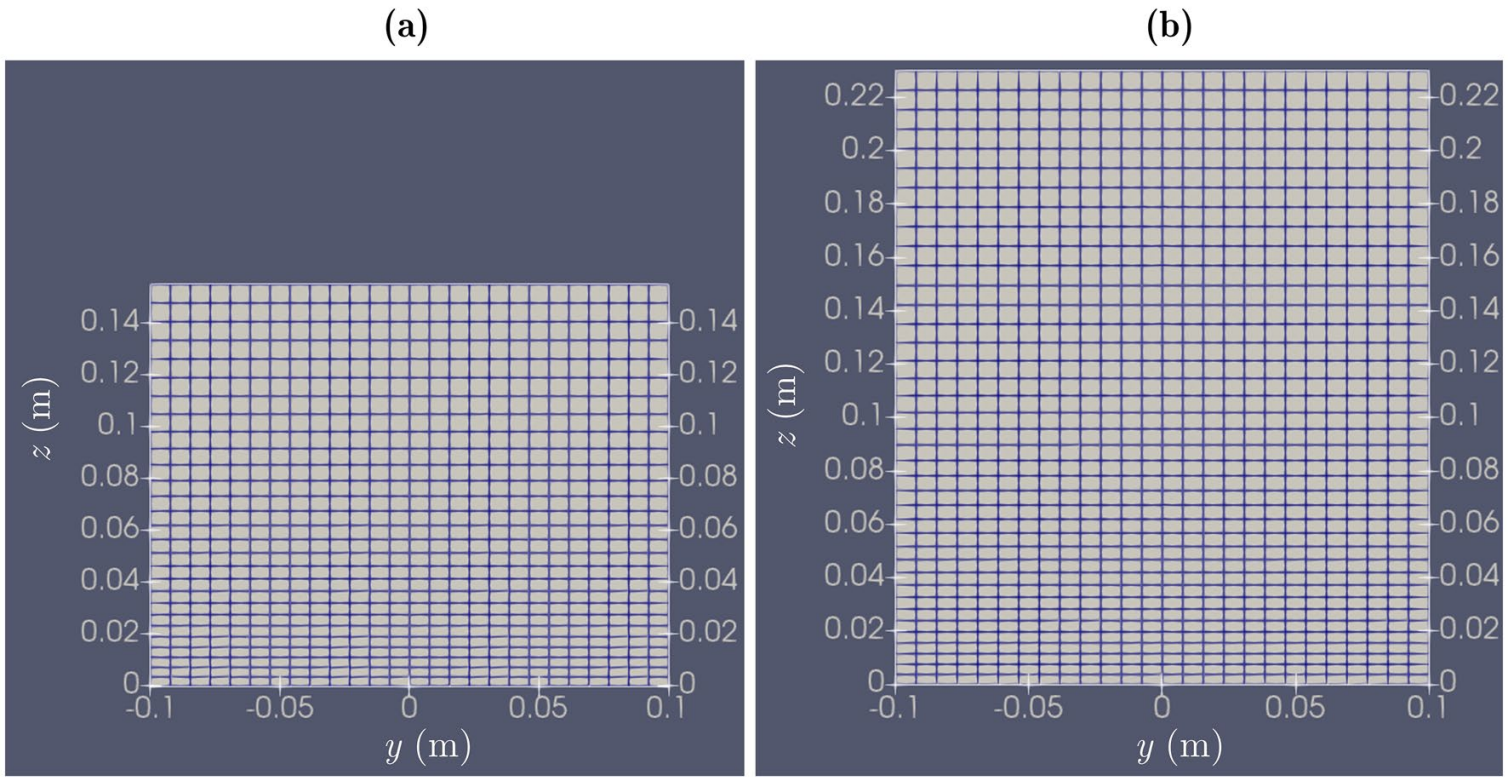
Cross-sectional mesh arrangements in the upstream part of the domain for: (**a**) Q45 and (**b**) Q70 [*z* = vertical distance from the bed and *y* = lateral position from the channel center].

In total, seven simulations were performed using OpenFOAM, developer version^44^, which is a 3D open-source CFD software based on the cell-centered finite volume method (FVM). The Q45, Q70, and Q95 simulation cases provided in Table 2 are used to compare the simulation results with the experimental and analytical results. Additionally, Q70_6mm and Q70_8mm cases were performed to check for grid convergence, and Q70_ke and Q70_koSST cases were performed to compare the results obtained for the *k–ε*^45^ and *k–ω* SST (shear stress transport)^46^ turbulence closure models, where *k* = turbulent kinetic energy, *ε* = dissipation rate of *k*, and* ω* = specific dissipation rate of *k*. Velocity–pressure coupling was achieved using the PIMPLE algorithm and the Reynolds-averaged Navier–Stokes (RANS) equations were closed using the renormalization group (RNG) *k–ε* model^47^. The simulations were performed using the *interFoam* solver, which captures the water–air interface using the volume of fluid (VOF) method. The water phase volume *α*_*water*_ = 0.5 corresponds to the free surface. The domain consists of three parts: 5.0 m and 1.0 m upstream and downstream straight parts and 5.352 m bend part mentioned in Table 2. A constant domain height of 0.23 m was used for *Q* = 0.07 m^3^/s and 0.095 m^3^/s, and the domain height was 0.155 m for *Q* = 0.045 m^3^/s, which saved simulation execution time. The computational mesh was generated using *blockMesh*, and the bend was defined using arc edges in the *blockMeshDict* dictionary. The cell sizes remained regular in the longitudinal direction (about 0.016 m for case numbers 1, 4–7, about 0.0125 m for case number 2, and about 0.02 m for case number 3) and lateral direction (about 0.0077 m for case numbers 1, 4–7, about 0.00625 m for case number 2, and about 0.0095 m for case number 3). However, in the vertical direction, smaller cells were used toward the bed to better represent the boundary layer. The near-bed cell heights are around 0.0037 m for case numbers 1, 4–7, about 0.003 m for case number 2, and about 0.0047 m for case number 3. These heights increase gradually toward the free surface and reach cell heights of about 0.0077 m for case numbers 1, 4–7, about 0.0061 m for case number 2, and about 0.0094 m for case number 3. At the first cell center, the obtained $z^{ + } = {{U_{*l} z} \mathord{\left/ {\vphantom {{U_{*l} z} \nu }} \right. \kern-0pt} \nu }$ values are 143, 154, and 176 for the Q45, Q70, and Q95 cases, respectively, where the laterally averaged shear velocity U_*l_ of the approach flow is calculated from the measured longitudinal velocity data using the log-law^48,49^ and *ν* = 1.09 × 10^–6^ m^2^/s at 16.6 °C. These *z*^+^ values higher than 30 satisfy the log-layer solution and justify the wall functions used^50^. The cross-sectional mesh arrangements for the Q45 and Q70 cases are shown in Fig. 3. The mesh arrangement for the Q95 case is very similar to that in Fig. 3(b). However, some minor differences are present due to the dissimilar inlet heights *h*_*in*_, which were measured during the experiments.

The inlet located 5.0 m upstream of the bend was assigned a *fixedValue* velocity condition with (*V*_*in*_ 0 0) as the value, where $V_{in} = {Q \mathord{\left/ {\vphantom {Q {bh_{in} }}} \right. \kern-0pt} {bh_{in} }}$ is assigned to the longitudinal component. As an initial condition, the domain was filled with water up to the depth = *h*_*in*_ using the *rotatedBoxToCell* region condition in the *setFieldsDict* dictionary in OpenFOAM, which helped to reduce the simulation execution time and to avoid instability. The outlet is a pressure-based outlet with *zeroGradient* condition for the basic parameters. For solid bed and sidewalls, *noSlip* velocity condition with standard wall functions were used for *k*, *ε* (or *ω*), and eddy-viscosity *ν*_*t*_^44^. The atmosphere at 0.23 m or 0.155 m height had a *pressureInletOutletVelocity* condition for the velocity, *totalPressure p0* = uniform 0 for the pressure, and an *inletOutlet* condition for *k*, *ε* (or *ω*), and *α*_*water*_. The maximum Courant number was set at 0.9. Each simulation was performed for 30 s. A local workstation computer with an Intel® Xeon® Gold 6248R CPU of 22 cores was used to run a simulation in parallel computing using the *scotch* method. The simulation execution times vary from 29 to 279 min, as provided in Table 2. The free surface undulation data were extracted using ParaView, version 5.9.1, and further analysis was performed using MATLAB.

## Results and discussion

### Grid convergence and selection of the turbulence closure model

**Figure 4:**
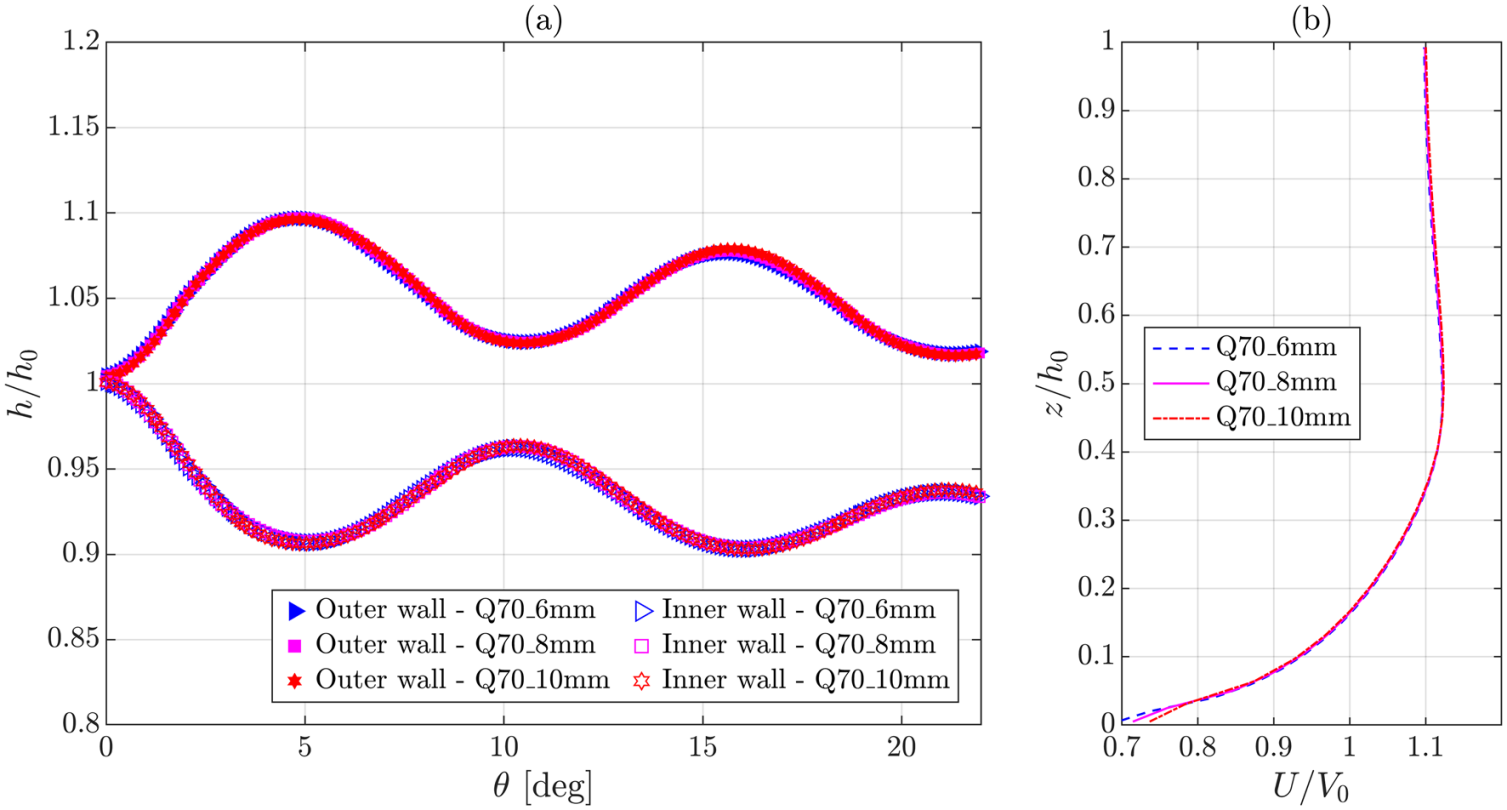
Comparison among the numerical results obtained for different grid arrangements at *Q* = 0.07 m^3^/s: (**a**) normalized WSPs along the outer and inner walls and (**b**) normalized longitudinal velocity profiles at the channel center located on the bend entrance plane [Q70_8mm is also used as case name Q70; see Table 2 for the legend details].

Figure 4a and b show the comparisons among the simulated WSPs along the outer and inner walls normalized as *h/h*_*0*_ and among the simulated normalized longitudinal velocity *U/V*_*0*_ profiles at the channel center located on the bend entrance plane, respectively, which were obtained for three different grid arrangements at *Q* = 0.07 m^3^/s, i.e., the first three cases provided in Table 2. Figure 4a and Table 3 indicate insignificant differences between the simulated WSPs and the simulated wave extrema water depths and their angular locations. Similarly, Fig. 4b signifies insignificant deviations between the simulated velocity profiles, except very close to the bed, which is unimportant in the context of the current study that does not focus on near-bed turbulence. The mean and maximum values of the absolute percentage change in *U/V*_*0*_ between the Q70_10mm and Q70_8mm cases are only 0.22% and 3.1%, respectively, and those between the Q70_8mm and Q70_6mm cases are only 0.16% and 2.9%, respectively, which are insignificant. Therefore, the simulation results converge for the tested grid arrangements. For the remaining four cases (Q70_ke, Q70_koSST, Q45, and Q95 in Table 2), the cell sizes are the same or comparable to those in case Q70.

**Table 3:** Comparisons among the first wave extrema results obtained from different grid arrangements and turbulence closure models.

Case name	*h*_*M*_ (m)	*θ*_*M*_ (deg)	*h*_*m*_ (m)	*θ*_*m*_ (deg)
Q70	0.1657	4.83	0.1371	5.11
Q70_6mm	0.1656 (− 0.06%)	4.89 (1.24%)	0.137 (− 0.07%)	5.22 (2.15%)
Q70_10mm	0.1655 (− 0.12%)	4.86 (0.62%)	0.1368 (− 0.22%)	5.03 (− 1.57%)
Q70_ke	0.166 (0.18%)	4.69 (− 2.9%)	0.1383 (0.88%)	4.83 (− 5.48%)
Q70_koSST	0.1648 (− 0.54%)	4.97 (2.9%)	0.1362 (− 0.66%)	5.11 (0.0%)

Note: Percent values indicate the deviations from the Q70 case results.

**Figure 5:**
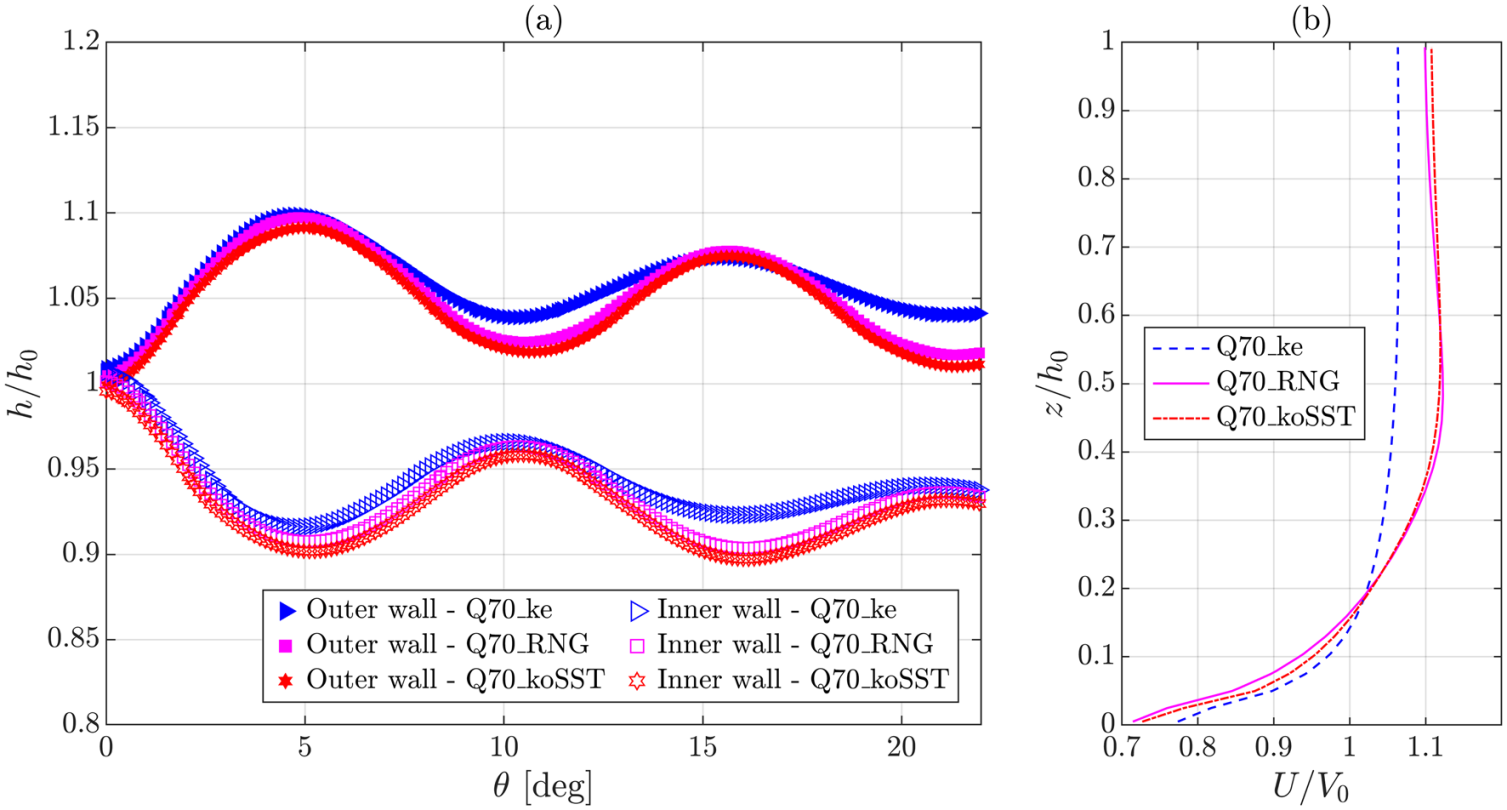
Comparison among the results obtained for different turbulence closure models at *Q* = 0.07 m^3^/s: (**a**) normalized WSPs along the outer and inner walls and (**b**) normalized longitudinal velocity profiles at the channel center located on the bend entrance plane [Q70_RNG is also used as case name Q70].

Figure 5a shows that the WSPs along the outer and inner walls computed using the RNG *k–ε* turbulence closure model are comparable to the WSPs simulated using the *k–ω* SST model. A similar trend is also observed for the *U/V*_*0*_ profiles, as shown in Fig. 5b. However, the *U/V*_*0*_ profile computed using the *k–ε* model deviates significantly from the other two profiles, possibly due to the difference in the model coefficients. Additionally, the WSPs obtained using the *k–ε* model deviate noticeably from the remaining two cases beyond the first wave extrema locations. Downstream of the first wave extrema, the *k–ε* model computes flatter wave profiles than do the other two models. No significant differences are observed for the first wave extrema water depths and their angular locations, as provided in Table 3. Therefore, the tested turbulence closure models do not significantly impact the WSP up to the first wave extrema location. Eventually, the RNG *k–ε* turbulence closure model was used for the remaining two discharges, 0.045 m^3^/s and 0.095 m^3^/s, i.e., case Q45 and Q95 in Table 2, which was previously found suitable for supercritical flows in a chute spillway by Huang and Wang^40^ and in curved channel models by Brown and Crookston^39^ and for subcritical flow in a curved channel model by Gholami et al.^51^.

### Comparison between the experimental, numerical, analytical, and empirical results

**Figure 6:**
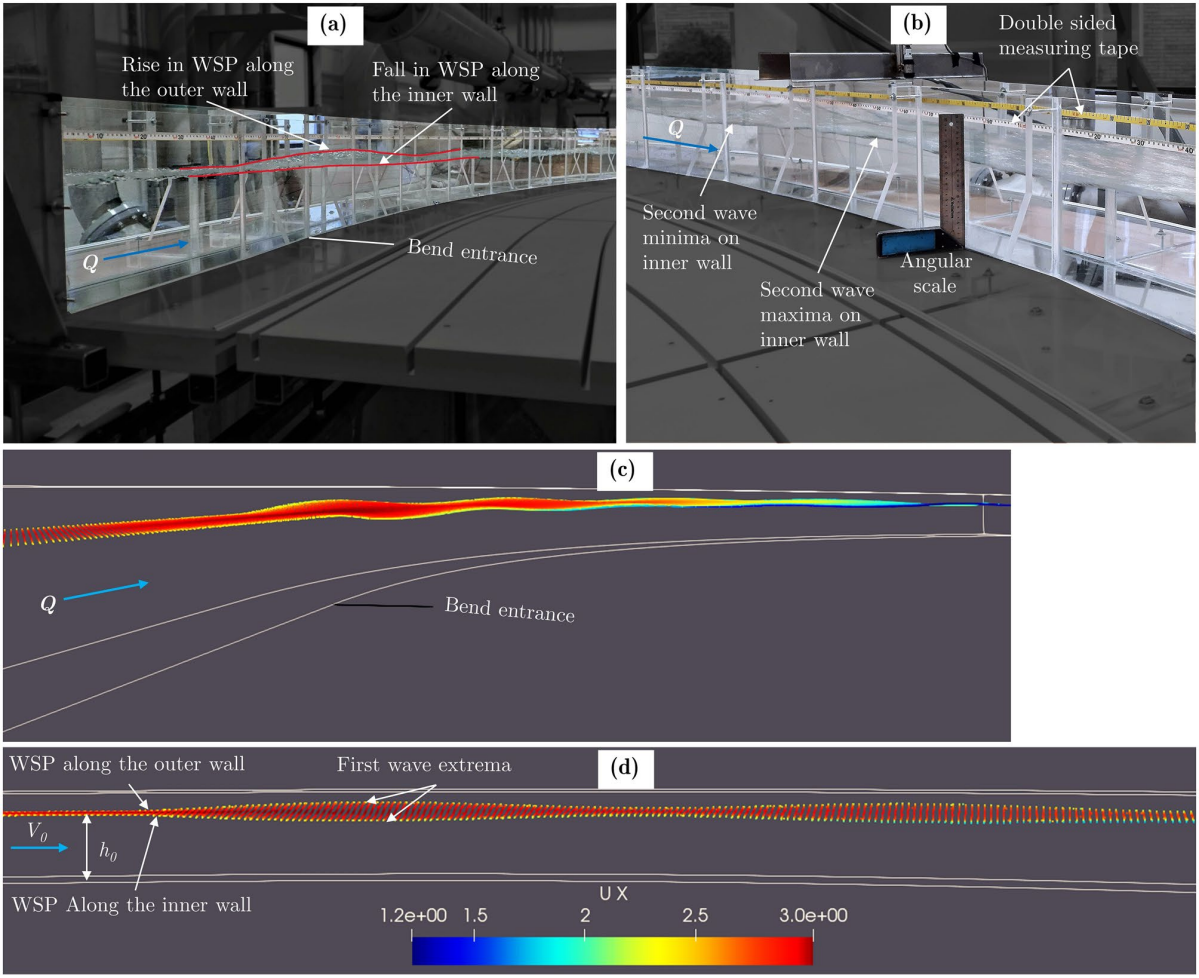
Experimental and simulated water surface undulations for *Q* = 0.095 m^3^/s: (**a**) 3D view of the observed undulations in the straight upstream part and in the channel bend [WSPs along the walls around the first wave extrema locations are marked in red], (**b**) observed undulations and WSP along the inner wall in the channel bend around the second wave extrema locations, (**c**) 3D view of the simulated undulations in the straight upstream part and in the channel bend, and (**d**) 2D view of the simulated WSPs up to second wave extrema locations, i.e., ≈ 4*θ*_*0*_ [*θ*_*0*_ = *θ*_*M*_ for the outer wall and *θ*_*0*_ = *θ*_*m*_ for the inner wall, U X = longitudinal velocity. See supplementary information for the video materials ©Kadia et al. (2024)].

The approach flow upstream of the bend entrance was found to be fairly-horizontal across the channel for both the experiments and simulations (see Fig. 6a and c, d for the results obtained for *Q* = 0.095 m^3^/s). The bend entrance acts as the point of disturbance to the flow, and the immediate downstream flow undulates across the channel and along the bend and follows the classical theory of cross-wave characteristics in supercritical flows^19,20,25^, as shown in Fig. 1. According to both the experimental and numerical results, the water surface rises along the outer wall as the positive wave front reaches the outer wall before reflecting toward the inner wall. Similarly, the negative wave front toward the inner wall drops the water surface along the inner wall. These phenomena continue until the first wave maxima and wave minima are reached. Further downstream, those wave extrema characteristics reverse and continue to alter (see Fig. 6) at angular intervals ≈ *θ*_*M*_ or ≈ *θ*_*m*_. Figure 6b shows the observed WSP along the inner wall for *Q* = 0.095 m^3^/s and the positions of the second wave minima and second wave maxima are marked, which are located at around *θ* ≈ 3*θ*_*m*_ and *θ* ≈ 4*θ*_*m*_, respectively.

**Figure 7:**
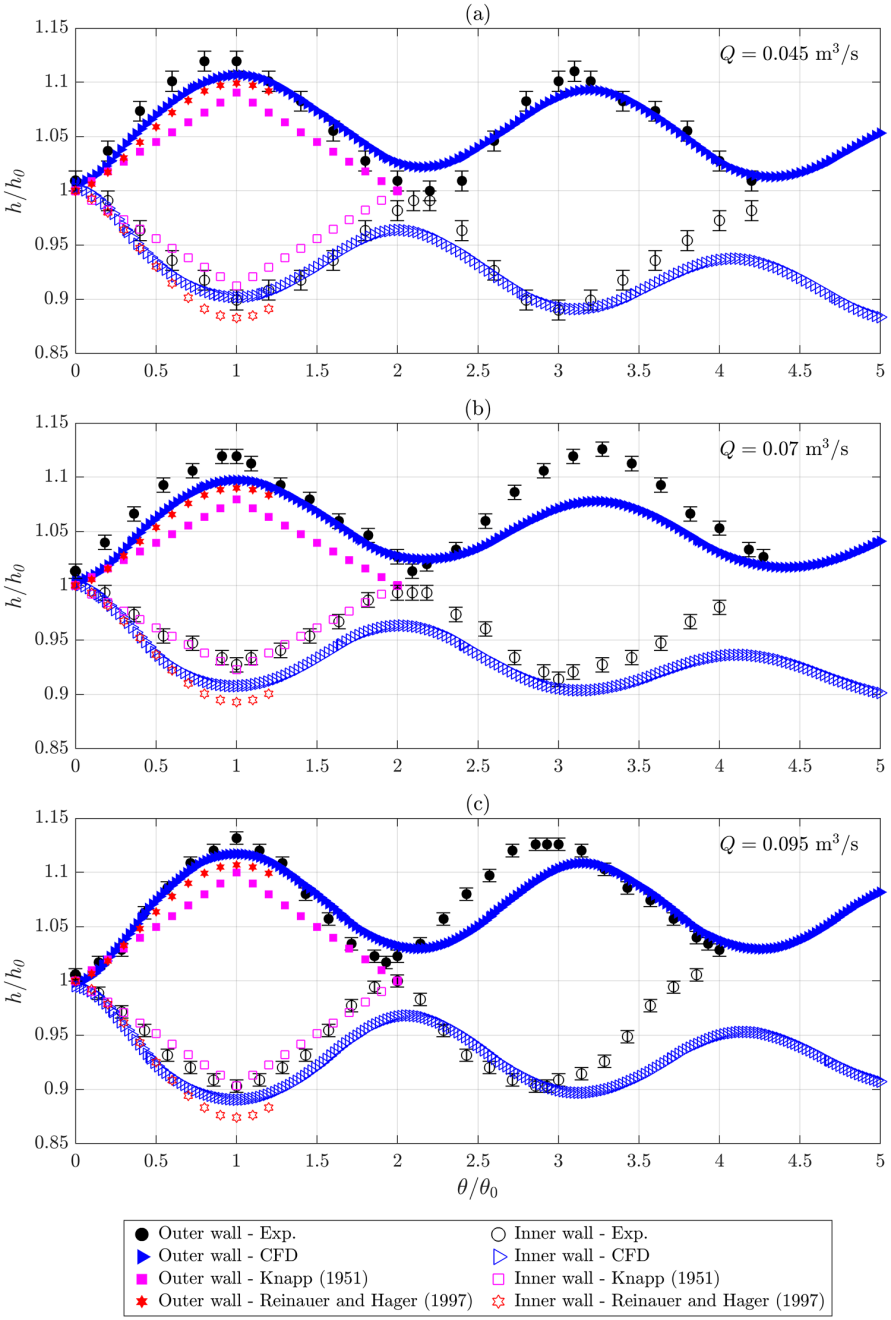
Comparison among the WSPs obtained experimentally, computationally, analytically, and empirically along the outer and inner walls for: (**a**) *Q* = 0.045 m^3^/s, (**b**) *Q* = 0.07 m^3^/s, and (**c**) *Q* = 0.095 m^3^/s [*θ*_*0*_ = *θ*_*M*_ for the outer wall and *θ*_*0*_ = *θ*_*m*_ for the inner wall; water level measurement uncertainties are provided using the error bars].

In Fig. 7, the simulated WSPs along the outer and inner walls are compared with the WSPs obtained experimentally, analytically, and empirically. Furthermore, Table 4 provides a comparison among the obtained first wave maximum and first wave minimum flow depths and their angular locations on the outer wall and the inner wall, respectively. Along the outer wall, the nondimensional flow depth *h/h*_*0*_ rises until the first wave crest located at *θ* = *θ*_*M*_ (provided in Table 4). Thereafter, it drops until the first wave trough positioned at *θ* ≈ 2*θ*_*M*_. These trends repeat in the downstream, as shown in Figs. 6 and 7. Along the inner wall, *h/h*_*0*_ drops until the first wave trough positioned at *θ* = *θ*_*m*_ (provided in Table 4) but rises thereafter until the first wave crest located at *θ* ≈ 2*θ*_*m*_. These phenomena subsequently repeat in the downstream. The simulated WSPs are consistent with the experimental data, especially up to *θ* ≈ 1.75*θ*_*0*_, as shown in Fig. 7. Furthermore, the simulated first wave extrema flow depths *h*_*M*_ and *h*_*m*_ deviate marginally (within ± 2.1%) from the observed ones as provided in Table 4. Slightly greater deviations, especially for the inner wall, are observed for Q70 than for Q45 and Q95, apparently due to some measurement uncertainties. Although the empirical profiles along the outer and inner walls obtained using Reinauer and Hager’s approach^20^ agree well with the simulated WSPs, the empirical WSPs are visibly lower than those observed from the experiments. The simulated WSPs around the wave trough for the outer wall and around the wave crest for the inner wall, i.e., for *θ* around 2*θ*_*0*_, are flatter than those observed experimentally. In addition, the analytical profiles obtained from Knapp’s approach^25^ look diamond shaped and deviate significantly from the simulated profiles obtained along the outer wall. This shape does not follow the sinusoidal profile because of *θ*_*0*_ <  < *β*_*w*_ (see Tables 1 and 4 and Eq. 1). The values of *h*_*M*_ and *h*_*m*_ obtained analytically and empirically deviate insignificantly (but more than the simulated results) from the observed data, within ± 3.6% and ± 3.7%, respectively (see Table 4).

**Table 4:** Comparisons among the first wave extrema water depths and their angular locations obtained from experiments, simulations, analytical model, and empirical model.

*Q* (m^3^/s)	*h*_*0*_ (m)	Experimental values		Simulated values		From analytical* approach		From empirical** approach	
		*h*_*M*_ (m)	*θ*_*M*_ (deg)	*h*_*M*_ (m)	*θ*_*M*_ (deg)	*h*_*M*_ (m)	*θ*_*M*_ (deg)	*h*_*M*_ (m)	*θ*_*M*_ (deg)
0.045	0.109	0.122	4.28	0.1206 (− 1.1%)	4.42 (+ 3.3%)	0.1189 (− 2.6%)	2.96 (− 31%)	0.1198 (− 1.8%)	3.47 (− 19%)
0.07	0.151	0.169	4.71	0.1657 (− 2.0%)	4.83 (+ 2.5%)	0.1630 (− 3.6%)	2.77 (− 41%)	0.1646 (− 2.6%)	3.31 (− 30%)
0.095	0.175	0.198	5.99	0.1955 (− 1.3%)	5.80 (− 3.2%)	0.1925 (− 2.8%)	3.10 (− 48%)	0.1937 (− 2.2%)	3.60 (− 40%)

		*h*_*m*_ (m)	*θ*_*m*_ (deg)	*h*_*m*_ (m)	*θ*_*m*_ (deg)	*h*_*m*_ (m)	*θ*_*m*_ (deg)	*h*_*m*_ (m)	*θ*_*m*_ (deg)
0.045	0.109	0.098	4.41	0.0983 (+ 0.3%)	4.69 (+ 6.3%)	0.0994 (+ 1.5%)	2.96 (− 33%)	0.0962 (− 1.8%)	4.90 (+ 11%)
0.07	0.151	0.14	4.85	0.1371 (− 2.1%)	5.11 (+ 5.4%)	0.1393 (− 0.5%)	2.77 (− 43%)	0.1348 (− 3.7%)	4.67 (− 3.7%)
0.095	0.175	0.158	6.18	0.1558 (− 1.4%)	5.94 (− 3.9%)	0.1581 (+ 0.1%)	3.10 (− 50%)	0.1530 (− 3.2%)	5.08 (− 18%)

Note: Percent values in the brackets indicate the deviations from the experimental results, *Knapp ^25^, **Reinauer and Hager ^20^.

Although the analytical and empirical WSPs plotted against the nondimensional angular position *θ/θ*_*0*_ (see Fig. 7) look comparable to the experimental and numerical results, the WSPs would shift significantly if plotted against the absolute *θ* due to trivial deviations between the analytical and empirical *θ*_*M*_ and *θ*_*m*_ values and the observed and numerical *θ*_*M*_ and *θ*_*m*_ values, as provided in Table 4. The analytical *θ*_*M*_ and *θ*_*m*_ values obtained from Knapp’s approach^25^ are up to 50% lower than the observed values, whereas the empirical *θ*_*M*_ values calculated using Reinauer and Hager’s approach^20^ are up to 40% lower than the observed values. The differences in the experimental conditions between the present study and Reinauer and Hager’s empirical study^20^, i.e., comparatively milder bend, narrower channel, and smaller bend number in the present study, have contributed to the observed deviations. Interestingly, the deviations observed for the empirical *θ*_*m*_ values obtained from Reinauer and Hager’s approach^20^ are lower than those found for *θ*_*M*_. Besides, the simulated *θ*_*M*_ and *θ*_*m*_ values deviate marginally (within ± 3.3% for *θ*_*M*_ and within ± 6.3% for *θ*_*m*_) from the experimental results. In addition, the experimental, numerical, and empirical^20^ solutions indicate that the location of the first wave minimum on the inner wall is positioned downstream of the location of the first wave maximum on the outer wall, i.e., *θ*_*m*_ > *θ*_*M*_. Furthermore, Table 4 reveals that these values increase considerably with the increasing *Q* for a constant *S*_*b*_.

Overall, the CFD model provides more precise computations of the WSPs along the sidewalls and more efficient predictions of the first wave extrema flow depths and their angular locations than do the available empirical and analytical approaches. Therefore, this open-source CFD model is useful in designing the height of hydraulic structures conveying supercritical bend flows. The marginal deviations obtained between the experimental and numerical results up to *θ* ≈ 1.75*θ*_*0*_ are partially attributed to the uncertainties associated with the measurement of the water surface using the angular scale (shown in Fig. 7) and discharge from the flowmeter. The maximum measurement uncertainty for the discharge is 0.42%, which includes a measurement accuracy of ± 0.2% (received from the calibration data) and a measurement least count of 0.0001 m^3^/s, which can be up to 0.22% [(0.0001 × 100%)/0.045]. Additionally, the 0.001 m measurement least count of the angular scale can result in a maximum uncertainty of 1.03% [(0.001 × 100%)/0.097, as the recorded minimum water depth is 0.097 m] in the water level measurements.

## Conclusions

This experimental and numerical study presents an open-source CFD model and investigates the water surface profiles along the outer and inner walls, the wave extrema flow depths, and their angular locations obtained for three supercritical flow conditions in a narrow open channel bend model (scale 1:22) of the Solis SBT from Switzerland using experiments, numerical simulation, and existing analytical and empirical approaches. The CFD simulations were performed using OpenFOAM, and the experiments were performed in the R&D Hydraulic Laboratory of Vattenfall AB. The flow conditions cover discharges *Q* = 0.045 m^3^/s, 0.07 m^3^/s, and 0.095 m^3^/s; approach flow depths *h*_*0*_ = 0.109 m, 0.151 m, and 0.175 m; aspect ratios *a*_*r*_ = 1.83, 1.32, and 1.14; Reynolds numbers Re = 3.95 × 10^5^, 5.12 × 10^5^, and 6.34 × 10^5^; and Froude numbers Fr ≈ 2. These *a*_*r*_ values are lower than those in previous studies listed in Table 1. The major conclusions drawn from the study are as follows:

- Although the water surface upstream of the bend is fairly-horizontal across the channel, it undulates in the bend due to cross-wave propagation. In the downstream of the bend entrance, the water surface rises along the outer wall and drops along the inner wall until reaching the respective wave crest and wave trough. The undulation pattern reverses after crossing the first wave extrema locations and continues to alter at angular intervals ≈ *θ*_*M*_ or ≈ *θ*_*m*_.
- The simulated WSPs are consistent with the experimental data, especially up to the angular location *θ* ≈ 1.75*θ*_*0*_. Furthermore, the simulated first wave extrema flow depths deviate marginally (within ± 2.1%) from the observed values. Such deviations are also lower for the simulated angular locations of the first wave extrema, within ± 3.3% for *θ*_*M*_ and within ± 6.3% for *θ*_*m*_. However, the simulated WSPs around the wave trough for the outer wall and around the wave crest for the inner wall, i.e., for *θ* around 2*θ*_*0*_, are flatter than those observed experimentally.
- Although the first wave extrema flow depths obtained analytically^25^ and empirically^20^ deviate insignificantly (but more than those found for the simulated results) from the observed data, within ± 3.6% and ± 3.7%, respectively, the deviations are significant for the angular locations of the first wave extrema, apparently due to the differences in the flow conditions. The analytical *θ*_*M*_ and *θ*_*m*_ values are up to 50% lower than the experimental data, while the empirical *θ*_*M*_ values are up to 40% lower. Therefore, although the analytical and empirical WSPs plotted against the nondimensional angular position *θ/θ*_*0*_ look comparable to the experimental and numerical results, the WSPs would shift significantly if plotted against the absolute *θ*.
- In addition, the location of the first wave minimum on the inner wall is positioned downstream of the location of the first wave maximum on the inner wall, i.e., *θ*_*m*_ > *θ*_*M*_, as obtained from the experiments, simulations, and empirical approach^20^. Furthermore, for a constant *S*_*b*_, those values increase considerably with an increasing discharge.
- Overall, the open-source CFD model computes the WSPs along the sidewalls, the first wave extrema flow depths, and their angular locations with better precisions than do the existing analytical and empirical methods. Therefore, this model is useful in studying the effects of a bend and resulting cross-waves on the water surface undulations and in producing accurate parameters useful in designing sidewall heights for hydraulic structures conveying supercritical bend flows.

## Future outlook

Presently, the turbulent flow characteristics and bulk sediment transport in the curved physical model of the Solis SBT are investigated using volumetric Particle Tracking Velocimetry (PTV) and high-speed cameras. In addition, the application of the presented open-source numerical model should be extended in the future to existing SBTs and other hydraulic structures conveying supercritical flows and to steep natural streams to further investigate the complex water surface undulations due to in-plan bends and to improve the existing analytical and empirical solutions (by producing additional data), which will facilitate better design of the height and angular location of retaining walls, embankments, levees, etc. those are used to protect floodplains.

### Supplementary Information

Supplementary Video 1.

Supplementary Video 2.

## Data Availability

The experimental and numerical results obtained from the study can be acquired from the corresponding author upon genuine request.

## Abbreviations

*a*_*r*_: *b/h* Or *b/h*_*0*_ = aspect ratio (-)
*b*: Channel width (m)
*B*_*n*_: Bend number (-)
*D*_*h*_: Hydraulic diameter (m)
Fr: Froude number (-)
*h*: Flow depth (m)
*h*_*M*_: First wave maxima flow depth on the outer wall (m)
*h*_*m*_: First wave minima flow depth on the inner wall (m)
*h*_*0*_: Approach flow depth (m)
*k*: Turbulent kinetic energy (m^2^/s^2^)
*Q*: Discharge (m^3^/s)
Re: Reynolds number (-)
*R*_*b*_: Radius of the bend (m)
*r*_*c*_: *b/R*_*b*_ = Relative radii of curvature (-)
*U*: Longitudinal velocity (m/s)
*V*_*0*_: Approach flow velocity (m/s)
*α*_*water*_: Water volume fraction (-)
*β*_*w*_: Wave angle (degree)
*ε*: Dissipation rate of turbulent kinetic energy (m^2^/s^3^)
*ν*: Viscosity of water (m^2^/s)
*ν*_*t*_: Eddy-viscosity (m^2^/s)
*ω*: Specific dissipation rate of turbulent kinetic energy (/s)
*θ*: Angular location in the bend (degree)
*θ*_*d*_: Angle of deviation (degree)
*θ*_*M*_: Angular location of the first wave maxima on the outer wall (degree)
*θ*_*m*_: Angular location of the first wave minima on the inner wall (degree)
*θ*_*0*_: Angular location of the first wave extrema (degree)
